# Key Chemical Soil Parameters for the Assembly of Rhizosphere Bacteria Associated with Avocado Cv Hass Grafted on Landrace Rootstocks

**DOI:** 10.1007/s00284-024-03917-0

**Published:** 2024-10-16

**Authors:** Mateo Córdoba-Agudelo, Juan C. Arboleda-Rivera, David A. Borrego-Muñoz, Camilo A. Ramírez-Cuartas, Juan E. Pérez-Jaramillo

**Affiliations:** 1 Instituto de Biología, Universidad de Antioquia, Medellín, Colombia; 2 Escuela de Microbiología, Universidad de Antioquia, Medellín, Colombia

## Abstract

Avocado cultivation holds significant economic importance in many countries, ranking Colombia as the fifth largest global producer. Particularly, the Hass cultivar plays a pivotal role in Colombia’s avocado industry, especially in the Department of Antioquia, the primary export region. This cultivar is grown under diverse soil and climate conditions and exhibits considerable genetic polymorphism due to the hybridization of varieties of agronomic significance, leading to a diverse array of landrace rootstocks. However, the role of soil conditions and rootstock genotype in structuring rhizosphere bacterial communities is still lacking. In addressing this knowledge gap, we investigated the influence of two soil conditions on the structure of rhizosphere bacterial communities associated with two landrace genotypes of *Persea americana* cv. Hass, utilizing 16S rRNA sequencing. Notably, no significant differences related to genotypes were observed. This study reports that the rhizosphere bacterial microbiome remains consistent across avocado landrace rootstocks, while variations in key parameters such as phosphorus, pH, Mg, and Ca drive distinct rhizosphere effects. Our results reveal that despite the soils having similar management, increases in these crucial parameters can lead to bacterial communities with lower alpha diversity and a more complex co-occurrence network. In addition, we found substantial variations in beta diversity, bacterial composition, and metagenome predictions between the two farms, underscoring the role of soil variables in shaping the bacterial microbiome. These findings provide valuable insights into the factors influencing the bacterial communities that may play a role in the health and productivity of crops with agro-industrial potential, such as Hass avocado.

**Supplementary Information:**

The online version contains supplementary material available at 10.1007/s00284-024-03917-0.

## Introduction

Plants interact with thousands of bacterial species throughout their life cycles [1]. In particular, the rhizosphere, the area of soil influenced by the roots, is the habitat for several bacterial groups that can readily use the exudates plants release into the soil, and in return, bacteria inhabiting the rhizosphere can help plants to effectively uptake nutrients, deal with water and saline stresses, and defend the plant against pathogens exerting an overall positive effect on plant growth [1]. In agricultural contexts, soil properties and management practices are undoubtedly the main drivers of bacterial diversity and community assembly in the rhizosphere [2]. However, a small but significant contribution comes from the plant side, where a plant-specific selection process is undertaken depending on plant species [3]. Furthermore, there is plenty of evidence that different plant genotypes, and even contrasting crop varieties of the same plant species, may recruit a particular set of microbial populations in the rhizosphere, which might be linked to genetic traits that as so far remain unknown [1, 3, 4]. The interactions between organisms in the rhizosphere have a significant impact on plant growth and health, playing a crucial role in agricultural sustainability. For instance, plant-associated microbiomes have received enormous attention because of their potential to help plants cope with biotic and abiotic stresses [5]. Current sequencing tools have made it possible to explore the composition, structure, and functional capabilities of microbial communities in agroecosystems, revealing that some microbial groups might exert biocontrol activities against pathogens [6]. Thus, the characterization of plant-associated bacterial communities as well as the biological interactions taking place in the rhizosphere is pivotal to untangle the mechanisms that influence plant growth and health [1].

In Colombia, the avocado industry has gained substantial prominence, currently ranking third in national fruit production, occupying approximately 7% of the country’s fruit cultivation area, and contributing to exports exceeding 30,000 tons in 2018 [7, 8]. Among avocado cultivars in Colombia, the Hass variety stands out as the most widely cultivated and exported, with an average annual yield of 10.8 tons per hectare. The Hass cultivar is renowned for its premium pulp quality, remarkable productivity, and late maturation [7]. In Colombia, avocado is grown across diverse climatic and soil conditions, giving rise to a plethora of landrace rootstock genotypes resulting from the hybridization of varieties of agronomic interest [8]. To optimize avocado production, a common agricultural practice involves grafting, where locally adapted rootstocks are combined with high-yielding clonal scions, such as the Hass cultivar [8]. Efforts to characterize the rootstocks of these landraces in Antioquia have been undertaken by Velázquez-Zapata et al. [9] and Reyes-Herrera et al. [10]. These studies revealed high genetic heterogeneity in the rootstocks of avocado cv. Hass in Antioquia independent of the sampled region. However, it remains a significant knowledge gap to ascertain whether these genotypes recruit distinct microbial communities and how these communities respond to varying soil conditions.

This work provides a detailed analysis of the rhizosphere bacterial communities of avocado cv. Hass using 16S rRNA sequencing, comparing two landrace rootstock genotypes in two farms with different soil conditions in the department of Antioquia. A robust experimental design was followed to determine how the physicochemical parameters of the soil and the genotype of the plant rootstock influence the diversity and structure of rhizosphere bacterial communities. We postulate that a thorough assessment of the effect of soil conditions and contrasting genotypes on the diversity and composition of bacterial communities in the rhizosphere might help identify the driving forces behind microbiome composition in real-field conditions for an economically relevant crop such as avocado.

## Materials and Methods

### Study Area

The study area consisted of two Hass avocado-producing farms located in the department of Antioquia, Colombia. The first farm, named “La Escondida” (LE), is in the eastern region of the department, in the municipality of Rionegro (6° 5′ 54.421'' N, 75° 26′ 12.965'' W) at an altitude of 2159.57 m.a.m.s.l. A second farm named “Cantabria de La Sierra” (CS), is in the North of the department, in the municipality of San Pedro de los Milagros (6° 29′ 39.652'' N, 75° 31′ 6.579'' W) at an altitude of 2422.64 m.a.m.s.l. Both farms implemented similar soil nutrient management practices [10]. In the two farms, two landraces avocado rootstock genotypes on cultivar Hass scion were grown, which were previously identified by Velásquez-Zapata et al. [9] using 13 microsatellites markers and were classified as Genotype 3 (G3) and Genotype 4 (G4). However, detailed information regarding the phenotypic traits of each genotype is currently lacking.

## Sampling and Physicochemical Analysis of Rhizosphere Soils

Rhizosphere soil samples were concurrently collected from the root systems of 8-year-old avocado trees in the productive stage at two distinct farms (CS and LE). A total of 32 samples were collected: 8 from G3 avocado trees and 8 from G4 avocado trees in each farm, for a total of 16 soil samples per farm and 32 considering those from both. The sampling process adhered to a completely randomized design, where 100 g of fine roots at a depth of 10 cm were extracted from four equidistant points around each tree, pooled (per tree), and subsequently stored at −20º C. Subsequently, the fine roots were vigorously shaken to release the bulk soil, and DNA was extracted from the root-attached soil, which was considered rhizosphere soil. In addition, this study included the measurement of various soil parameters, including soil protein content, active soil carbon (permanganate oxidizable carbon), soil respiration, and soluble phosphorus. The methods employed for these analyses included the Bradford protein assay, potassium permanganate oxidation, KOH method, and phosphorus adsorption isotherm with CaCl_2_⋅2H_2_O (0.01 M), respectively. Furthermore, the content of major and minor elements was determined by a certified laboratory, and complementary climatic data were sourced from local meteorological stations (Table S1).

## DNA Extraction, Sequencing, and Bioinformatic Analysis

Soil DNA extractions were carried out using the DNeasy® PowerSoil commercial kit (QiagenTM) following the manufacturer’s specifications. The extracted DNA’s purity and concentration were validated using a NanoDrop1000, while its integrity was assessed via 1% agarose gel electrophoresis. DNA concentrations were adjusted to 50 ng/μL, as recommended by the external sequencing service. The PCR amplification of the 16S rRNA gene, library construction, and sequencing of the extracted DNA were conducted at the facilities of Macrogen, Inc. in Korea, following standard specifications. The 16S rRNA V3-V4 regions were amplified by using specific primers (Forward primer = 5′CCTACGGGGNGGCWGCAG-3′ and Reverse primer = 5′GACTACHVGGGTATCTAATCC-3′). For the PCR amplification, the reaction mixture was prepared as follows: 2.5 µL of microbial DNA (5 ng/µL), 5 µL of forward primer (1 µM), 5 µL of reverse primer (1 µM), and 12.5 µL of 2 × KAPA HiFi HotStart ReadyMix was added. The amplification was carried out using a thermal cycler with the following program: an initial denaturation at 95 °C for 3 min, followed by 25 cycles of denaturation at 95 °C for 30 s, annealing at 55 °C for 30 s, and extension at 72 °C for 30 s. A final extension at 72 °C for 5 min was performed, and the reaction was held at 4 °C. Amplicon libraries were constructed by using the Nextera XT Index Kit. Sequencing was performed by Illumina Miseq, with a maximum read length of 301 base pairs, and demultiplexed sequences were obtained for all 32 samples.

Bioinformatic processing was carried out using QIIME2 version 2020.2.0 [11]. Briefly, sequence quality was assessed, and based on the results, truncation lengths were set to 270 base pairs for the forward reads and 230 base pairs for the reverse reads. The sequences were then merged and clustered into amplicon sequence variants (ASVs) using the DADA2 plugin with default parameters applied for correcting substitution and indel errors, as well as chimera removal [12]. Taxonomic assignment was performed using the Naive Bayes classifier from sci-kit-learn, 16S rRNA V3-V4 primers, and the SILVA 132 database [13, 14]. To ensure the normalization of sampling efforts, sequence rarefaction was applied, adjusting to the sample with the lowest sequencing depth.

## Diversity of the Rhizosphere Bacterial Communities

All the downstream data analysis was conducted using the microeco R package version 0.3.3 [15, 16]. To assess alpha diversity, Chao 1, Faith, Pielou, and Shannon indices were computed. Bray–Curtis and Weighted Unifrac dissimilarity matrices were calculated based on the ASVs table to evaluate beta diversity [17]. Subsequently, Principal Coordinate Analysis (PCoA) was performed for each distance.

## Differential Abundance Analysis and Construction of Co-occurrence Networks

A Random Forests analysis was implemented to identify families, and genera with differential abundance between farms, utilizing the microeco R package. To gain insights into microbial community interactions, explore modules, and identify taxa associations, the microeco package was employed to create co-occurrence networks. The resulting co-occurrence networks were subsequently visualized using Gephi [18].

## Ordination Analysis and Functional Prediction

To assess the influence of soil variables on the structure of rhizosphere bacterial communities, a distance-based redundancy analysis (db-RDA) was conducted using Bray–Curtis distances. Finally, a functional prediction was conducted utilizing the PICRUSt2 plugin within QIIME 2, employing the taxonomic information derived from the ASV table [19]. The resultant pathways were further categorized within the MetaCyc categories associated with the synthesis and degradation of compounds [21].

## Statistical Analysis

All statistical analyses were performed using R version 4.3.3. Differences in farm conditions were assessed with the Wilcoxon test at a significance level of α = 0.05. Kruskal–Wallis tests (α = 0.05) were applied to compare alpha diversity indices between farms and genotypes. Beta diversity significance was evaluated through PERMANOVA using 999 permutations, with a significance threshold of *α* = 0.05. For differential abundance, Random Forest analysis was performed with 1000 trees and FDR-adjusted p values at an alpha level of 0.05. Co-occurrence networks were constructed using Spearman’s correlation analysis of ASVs, with a correlation threshold of 0.7 and *p* < 0.05. db-RDA analysis was conducted only on variables that showed significant differences between farms and contributed significantly to the model (*p* < 0.05). Finally, PICRUSt2 predictions were compared between farms using ALDEx2 with default parameters [20], and the output was filtered using the Benjamini–Hochberg correction (*p* < 0.01).

## Results

### Differences in Variables Associated with Soil, Climate, and Avocado Production

Paired comparisons were conducted for the assessment of 42 soil, yield, and climate variables. Notably, 19 variables exhibited statistically significant variations between the two farms (*p* < 0.05). Specifically, soil attributes such as exchangeable Ca and Mg were notably higher in LE, with values of 654 mg/kg and 126 mg/kg, respectively, in contrast to CS, where the obtained values were 228 mg/kg and 51.6 mg/kg, respectively. Conversely, variables like exchangeable acidity and available Fe content were 1.18 cmol/kg and 312.44 mg/kg in CS, and 0.28 cmol/kg and 85.3 mg/kg, in LE, respectively. Several other soil parameters displayed significant differences, including organic matter content, P, electrical conductivity, pH, available Cu, active carbon levels, and soil protein content (Table 1). Factors associated with avocado production, such as the number of avocados produced per tree, the number of avocados exported per tree, and the weight of avocados per tree (in grams), exhibited higher values in LE (260.6, 348.78, and 54,669.63, respectively) in contrast to CS (165.83, 142.63, and 30,028.66, respectively). Remarkably, the only climatic variable displaying a significant difference was the average ambient temperature. In CS, the average ambient temperature recorded was 16.08° C, while in LE, it was notably higher at 17.7° C (Table 1).

**Table 1:** Evaluation of climatic, yield, and soil variables in farms CS and LE. Wilcoxon tests were employed to assess differences between the two farms. The table exclusively displays variables that exhibited significant differences (*p* < 0.05). A comprehensive list of all measured variables is available in Table S1

Variable	CS		LE	
	mean	sd	mean	sd
Active carbon (mg/kg)	1314.55	99.55	1171.51	110.5
Aluminum Saturation (%)	0.255	0.12	0.02	0.01
Average Avocado Export per Tree (avocado/harvest)	142.63	69.12	345.78	243.33
Average Avocado Production per Tree (avocado/harvest)	165.83	82.14	245.1	276.97
Average Avocado Weight Exported per Tree (grams/harvest)	30,028.66	14,313.31	54,669.63	39,213.07
Calcium Saturation (%)	0.38	0.1	0.65	0.1
Copper Availability (mg/kg)	1.89	0.68	3.83	1.49
Electrical Conductivity (dS/m)	0.03	0.01	0.435	0.17
Exchangeable Acidity (cmol/kg)	1.18	0.54	0.42	0.27
Exchangeable Aluminum (cmol/kg)	0.77	0.35	0.35	0.26
Exchangeable Calcium (mg/kg)	228	78	654	258
Exchangeable Magnesium (mg/kg)	51.6	27.6	126	43.2
Iron Availability (mg/kg)	312.44	149.19	85.3	8.66
Log of Aerobic Endospore-Forming Bacteria	6.596	6.952	11.529	11.43
Magnesium Saturation (%)	0.14	0.04	0.21	0.03
Mean Ambient Temperature (°C)	15.97	2.07	17.58	1.6
Organic Matter (%)	10.63	3.77	15.09	2.88
pH	5.17	0.2	5.35	0.16
Phosphorus Availability (mg/kg)	2.69	0.43	5.71	1.83
Soil Protein (mg/L)	179.83	19.81	240.59	50.47

## Sequencing and Taxonomic Features

Rhizosphere soil DNA was successfully extracted and sequenced from 32 samples, yielding a collection of 2,426,188 demultiplexed sequences, with an average of 75,818 sequences per sample. Quality control measures and sequence merging procedures led to the identification of 15,427 amplicon sequence variants (ASVs), which collectively appeared 470,020 times across the 32 samples (Table S2). To ensure uniformity in sequencing depth and the reliability of alpha diversity assessments, rarefaction was performed at a standardized sequencing depth of 11,086 sequences per sample. This approach was validated through rarefaction curves (Fig.S1). After rarefaction, 354,720 reads were retained, accounting for 75.47% of the initial dataset.

**Fig. 1:**
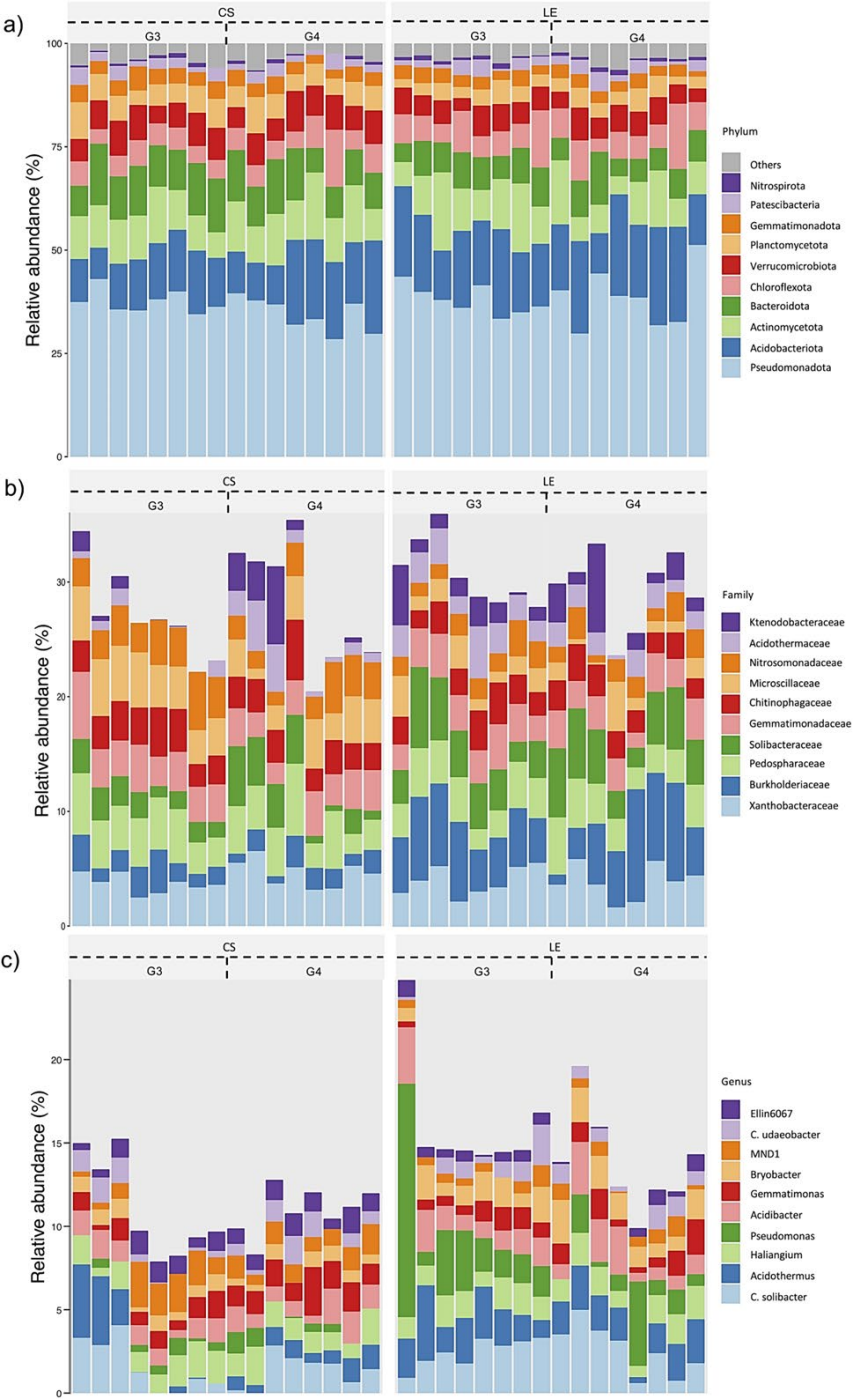
Taxonomic composition of the avocado rhizosphere of 32 samples in two producing farms with two landraces rootstocks genotypes. The top 10 most abundant taxa are displayed, and the rest are assigned to “Others.” **a** Phylum level. **b** Family level. **c** Genus level

The rhizosphere bacterial communities associated with the sampled avocado roots were characterized by a predominant representation of specific phyla. Notably, Pseudomonadota, Acidobacteriota*,* and Actinomycetota collectively accounted for over 50% of the relative abundance in each sampled root (Fig. 1a). In addition to these dominant phyla, other prevalent phyla included Bacteroidota*,* Chloroflexota*,* and Verrucomicrobiota. At higher taxonomical levels, it was shown that *Xanthobacteraceae, Burkholderaceae, Pedospharaceae,* and *Solibacteracae* families played a significant role in shaping the bacterial community composition (Fig. 1b, Table S3). Further exploration at the genus level revealed key contributors to the bacterial community abundance in the rhizosphere of Hass avocado trees. Genera such as *Candidatus Solibacter*, *Acidothermus*, *Haliangium*, *Pseudomonas*, and *Acidibacter* were identified as significant components of the microbial community (Fig. 1c, Table S3).

## Location Is the Main Driver of Rhizobacterial Diversity

**Fig. 2:**
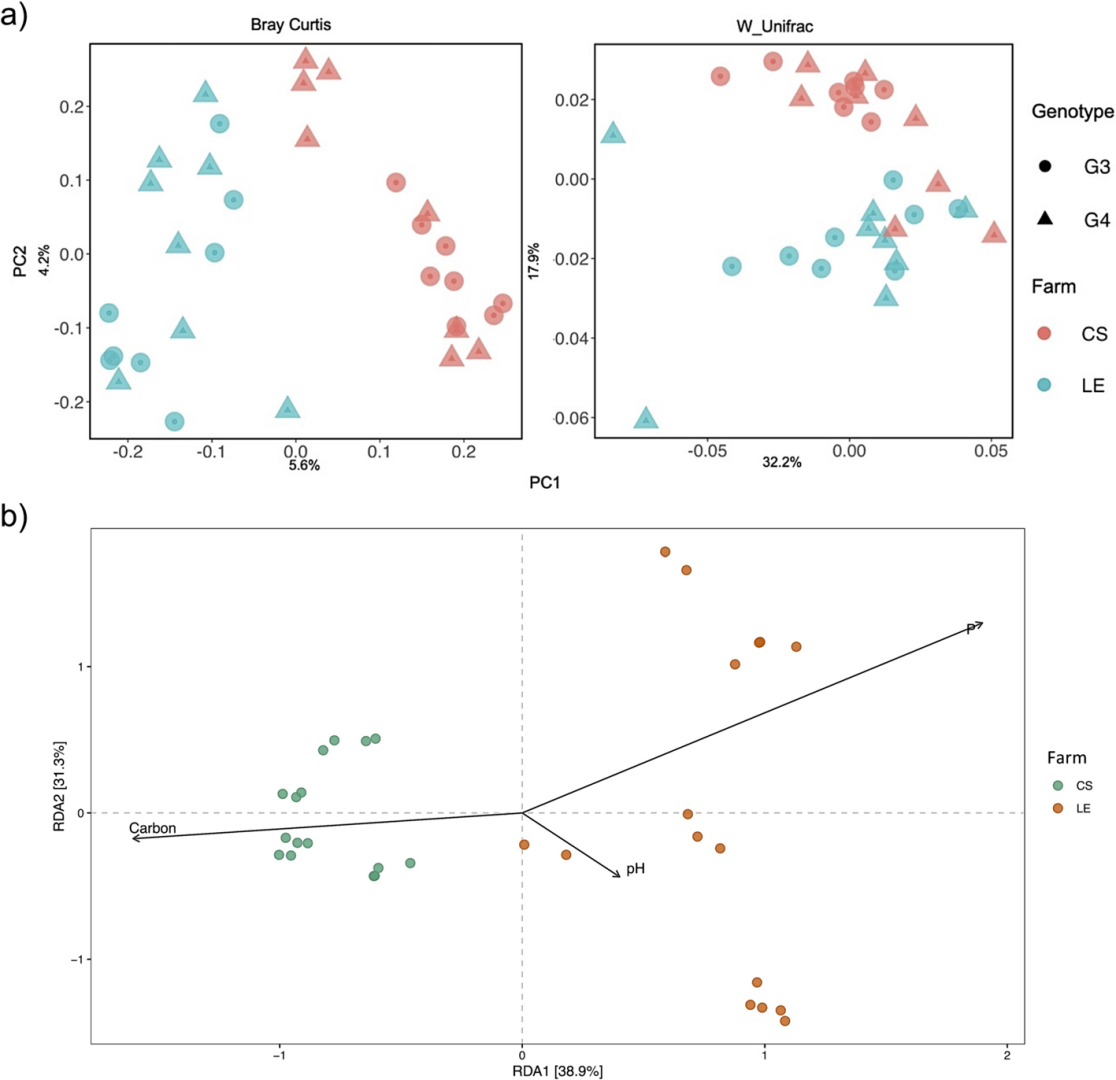
**a** Principal coordinate analysis (PCoA) of bacterial communities based on Bray–Curtis (left) and Weighted Unifrac distances (right). **b** Distance-based Redundancy Analysis (db-RDA) of the relationship between soil factors and bacterial community structure in samples from CS (green) and LE (orange). Legends: Carbon – active soil carbon; P – Phosphorus availability (Color figure online)

The Kruskal–Wallis test showed significant differences in the Shannon and Pielou indexes between farms (*p* < 0.05), with higher diversity observed in CS. However, the effect size is small. In contrast, no significant differences were observed in any of the alpha diversity indexes when comparing different genotypes (*p* > 0.05) (Fig. S2). The structure of bacterial communities across all samples was assessed using the Bray–Curtis and Weighted Unifrac distances, revealing clear structuring between farms but not among different genotypes (Fig. 2a, Fig. S3). To further investigate the bacterial community structure, a separate analysis was conducted for each farm (Fig. S4). CAP analysis suggests a distinction between genotypes within each farm, though this distinction did not reach statistical significance (CS, *p* = 0.446; LE, *p* = 0.389). These results robustly indicate that the variations in microbiome composition are primarily associated with farms rather than genotypes.

## Edaphic Factors Are the Main Drivers of Community Structure

To assess the impact of soil variables on the structuring of rhizosphere bacterial communities, a Distance-based Redundancy Analysis (db-RDA) was conducted for the farms. The first and second axes explained 38.9% and 31.3% of the total variance, respectively. Significant factors influencing the bacterial community structure, in order of importance, are phosphorus availability, active carbon, and pH. These variables displayed unique influence patterns suggesting that they exert specific effects on the bacterial communities in each farm (Fig. 2b).

## Taxa Relative Abundance Shows Dependence on the Farm

A Random Forest analysis was applied to identify specific bacterial families and genera with varying relative abundances between the two farms in total; 23 families exhibited differential representation, with 14 families showing higher prevalence in the CS farm, while for LE farm, 9 families showed enrichment. In the CS farm, overrepresented families included those belonging to Pseudomonadota *(Hyphomicrobiaceae, Dongiaceae, Nitrosomonadaceae, Halieaceae),* Bacteroidota *(Microscillaceae),* Actinomycetota *(Solirubrobacteraceae, Microbacteriaceae, Iamiaceae),* Planctomycetota *(Phycisphaeraceae, Pirellulaceae),* Bacillota *(Erysipelotrichaceae),* Mixococcota *(Sandaracinaceae), Campylobacteriota (Hydrogenimonadaceae),* and Chloroflexota *(Caldilineaceae)* phyla. Conversely, the LE farm showed an enrichment of families primarily associated with the Acidobacteriota *(Solibacteraceae, Holophagaceaea),* Pseudomonadota *(Methyloligellaceae, Pseudomonadaceae, Moraxellaceae, Burkholderiaceae, Rhodanobacteraceae),* and Actinomycetota *(Acidothermaceae, Nocardiaceae)* phyla.

**Fig. 3:**
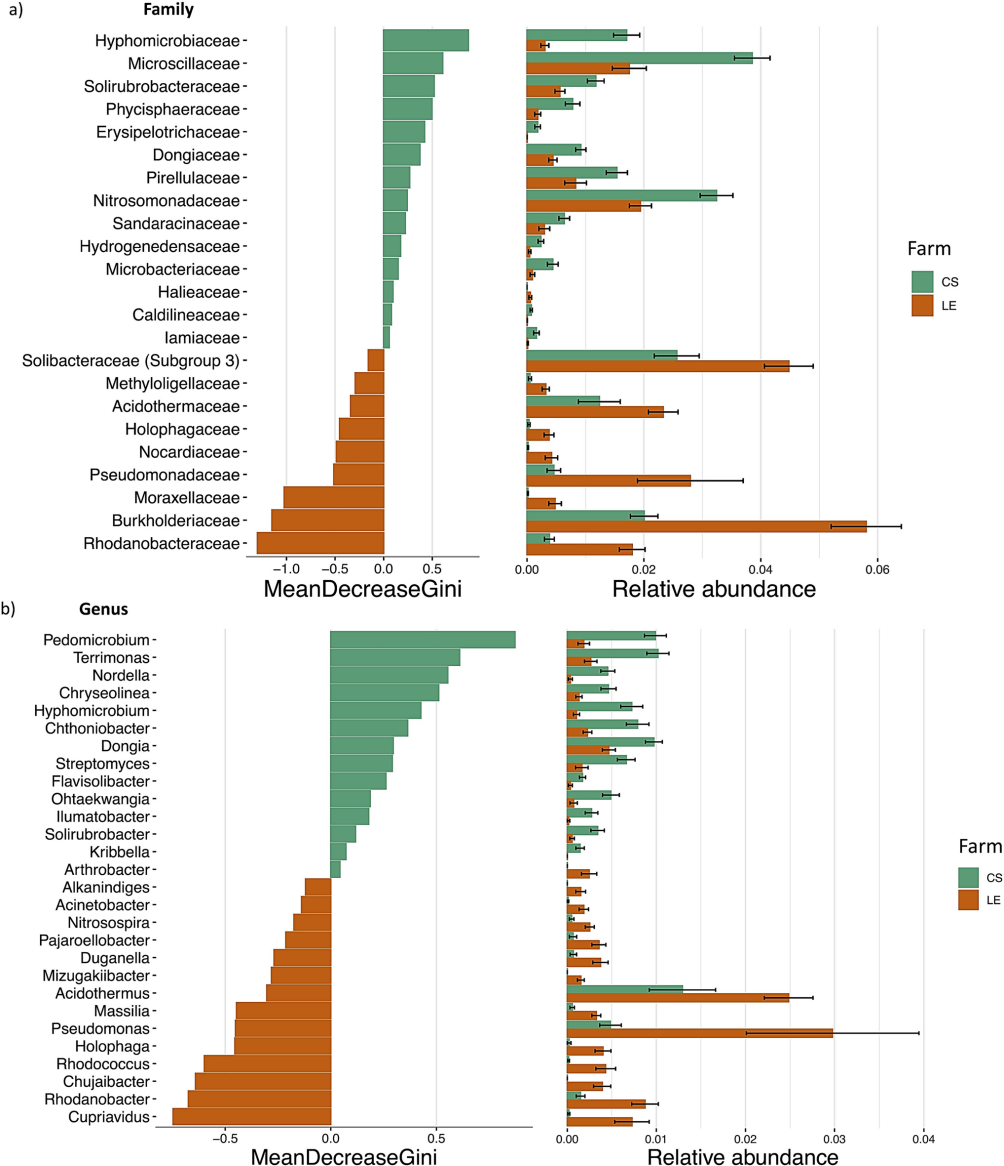
Random-forest classification of the relative abundance of avocado rhizosphere bacterial communities in landrace varieties across two farms at family (**a**) and genus (**b**) level. Left: Taxa are presented in descending order of importance, about their contribution to the model’s accuracy for each farm. Right: Relative abundance of taxa enriched in each farm. The bars represent the mean relative abundance, and error bars denote the standard error

At the genus level, CS exhibited an enrichment of several genera, including *Pedomicrobium, Terrimonas, Nordella, Chryseolinea, Hyphomicrobium, Chthoniobacter, Dongia, Streptomyces, Flavosolibacter, Ohtaekwangia, Ilumatobacter, Solirubrobacter, Kribbella,* and *Arthrobacter*. In contrast, LE demonstrated an enrichment of genera such as *Alkanindiges, Acinetobacter, Nitrospira, Pajeroellobacter, Duganella, Mizagukiibacer, Acidothermus, Massilia, Pseudomonas, Holophaga, Rhodococcus, Chujaibacter, Rhodanobacter,* and *Cupriavidus* (Fig. 3).

## Higher Co-occurrence Network Complexity in LE Soil

**Fig. 4:**
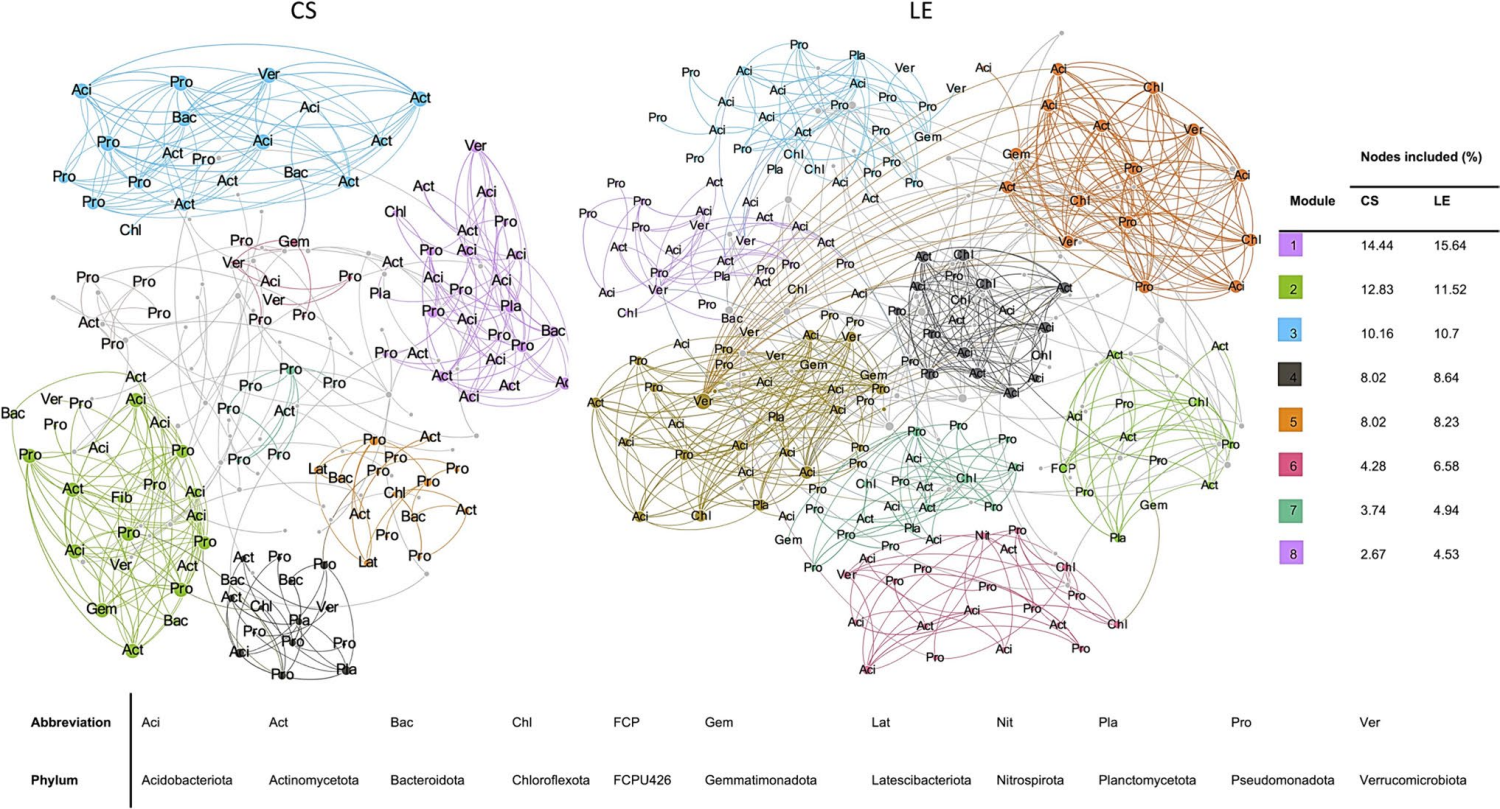
Analysis of co-occurrence patterns in bacterial communities within each farm (left: CS, right: LE). The relationships between ASVs were established using Spearman correlation, with a correlation coefficient threshold (ρ) of |0.7| and a significance level of *p* < 0.05. Nodes and edges were randomly colored according to the top 8 modules. Phyla abbreviations are displayed for the nodes within the top modules, while other nodes are depicted in light gray without labels. Node size is proportional to the node degree (Color figure online)

The co-occurrence patterns of rhizosphere bacterial communities associated with two farms were analyzed using Spearman correlations. In both networks, the nodes with higher degrees were composed of Pseudomonadota, Actinomycetota, and Acidobacteriota phyla. For CS, a network with 14 modules, 187 nodes, and 323 edges was identified, and Pseudomonadota*,* Planctomycetota, and Acidobacteriota had the highest Betweenness Centrality values. On the other hand, for LE, a network with 28 modules, 243 nodes, and 611 edges showed more variability. The LE network was more complex, with higher modularity, number of nodes, and edges compared to the CS network. For CS network, ASVs belonging to the *Solirubrobacteraceae* family exhibit a maximum degree of 12, while for LE, ASVs from the Pedosphaeraceae family have a maximum degree of 20. The co-occurrence modules in CS are primarily composed of families such as *Xanthobacteraceae, Burkholderaceae,* and *Microscillaceae*. In contrast, the modules in LE are composed of families like *Burkholderaceae, Pseudomonadaceae,* and *Xanthobacteraceae* (Fig. 4).

## Metagenome Prediction suggests a Rhizodeposit-consuming Microbiome

**Fig. 5:**
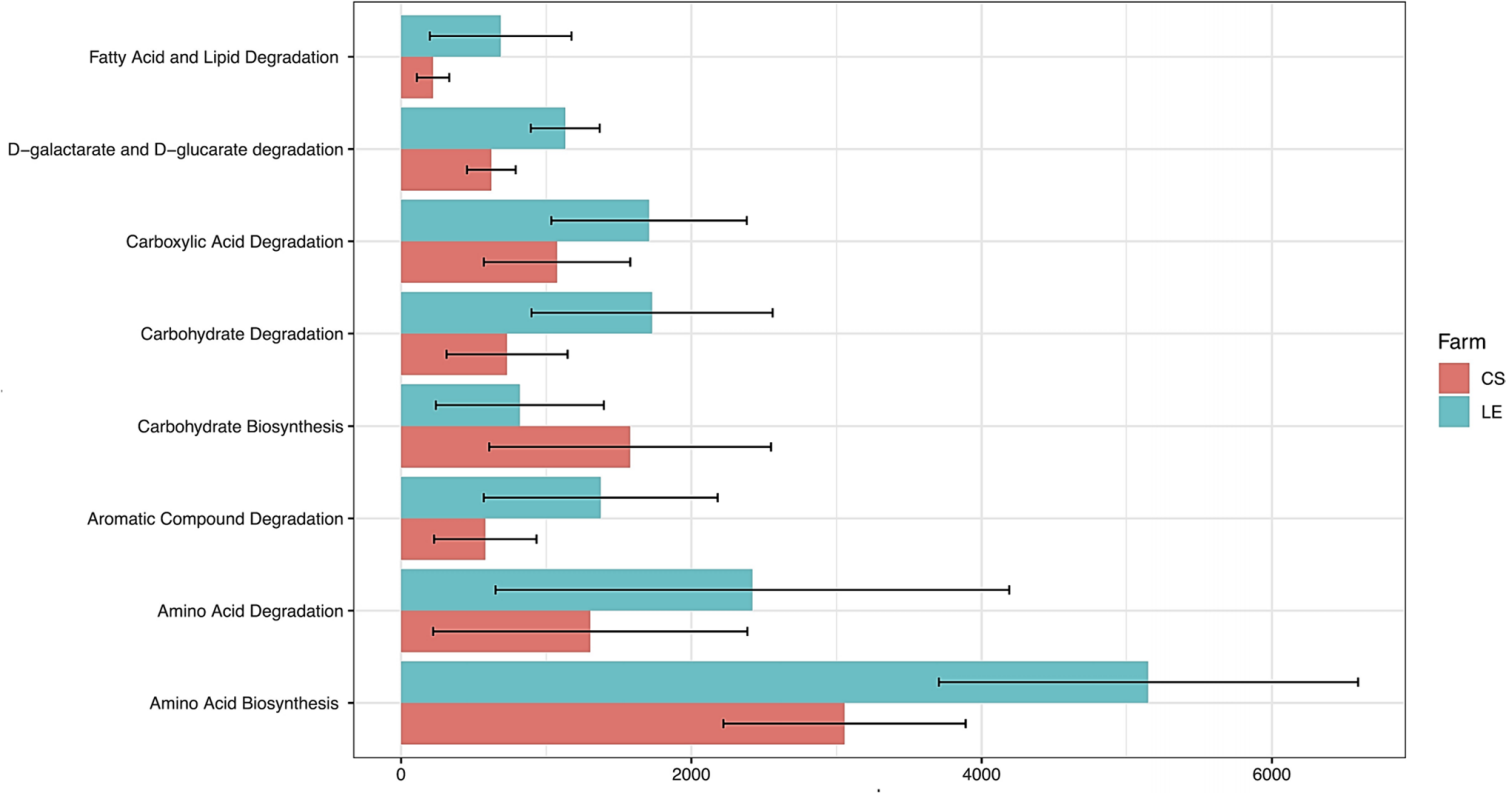
Prediction of the functional profile in the rhizosphere of avocado landraces across two farms, with data collapse by Metacyc pathway families. Aldex2 analysis was utilized to identify pathways with a significant presence in the rhizosphere microbiome of each farm (*p* < 0.01)

The functional profile of rhizosphere bacterial communities was predicted using PICRUSt2. Aldex2 analysis unveiled significant differences in 8 pathways between the farms. Notably, Carbohydrate Biosynthesis was the sole pathway category that exhibited higher abundance in CS, while pathways linked to degradation (carbohydrates, carboxylic acid, fatty acid, galactarate, glucarate, lipid, amino acid, and aromatic compounds) were more pronounced in LE. Also, amino acid biosynthesis pathways were more abundant in LE (Fig. 5).

## Discussion

This study presents a robust characterization of the bacterial communities associated with two landrace avocado rootstocks in two farms. While avocado rhizosphere microbiome has gained attention in recent years, to the best of our knowledge, this research is the first to report on the bacterial communities associated with avocado landrace rootstocks. Our findings reveal homogeneity in the bacterial communities between the rhizospheres of the two landrace rootstocks. Notably, we observed no significant differences in bacterial composition, alpha diversity, or beta diversity between these rootstocks. While genotypic characterization has revealed differentiation among landrace rootstocks [10], the Hass scion effect, or a comprehensive phenotypic characterization that includes traits such as height and weight, avocado production, fruit quality, and resistance to soil-borne pathogens is currently lacking. In addition, key factors in shaping the rhizosphere, such as root exudates remain uncharacterized for both genotypes.

Although the two soils exhibit similar edaphic conditions, we have identified significant differences in key soil variables critical for plant growth, such as pH, phosphorus, magnesium, and calcium. These key changes could enhance avocado nutrition, which is reflected in the higher avocado production in the LE soil. The correct soil conditions also play a pivotal role in shaping the rhizosphere microbiome structure and the intricate relationship with plants. Nutrient deficiencies impact the quality and quantity of root exudates and, when plants have an adequate supply of nutrients, their exudates can enhance beneficial plant–microbe interactions, creating a beneficial cycle for the plant [22]. Recent evidence has demonstrated that under sufficiently nutritious conditions, plants take the initiative in establishing beneficial plant–microbe associations [23]. In our study, we observed specific patterns of the bacterial communities in two soils with key differences, which may serve as an indicator of supportive soil properties for microbial development and, at the same time, indicate a strong rhizosphere effect driven by the plant [24, 25].

In terms of alpha diversity, LE showed a lower value compared to CS. Other studies have also reported that soils with greater availability of macronutrients have lower diversity in their bacterial communities than less fertile soils [26]. In this context, Hanley et al. [27] showed that bacterial communities are less diverse in nutrient-enriched soils; however, when plants are transplanted into nutrient-lacking soils, the communities exhibit increased diversity. The exudation patterns of plants are a fundamental trait that can significantly influence the establishment of bacterial communities and can undergo alterations in response to low-nutrient concentrations, consequently impacting the bacterial communities [28, 29]. While we currently lack evidence to link the differences in microbial communities to varying exudation patterns, we propose that future studies should prioritize investigating this hypothesis.

At a phylum taxonomic rank, the rhizosphere microbiome is predominantly composed of phyla such as Pseudomonadota, Acidobacteriota, and Actinomycetota. These findings are consistent with previous reports on the avocado rhizosphere microbiome, where these phyla were identified as dominant components [30]. At the genus level, there is more variability observed between studies; however, *Pseudomonas* emerges as a ubiquitous genus [30]. It has been reported that *Pseudomonas* spp. can establish intricate relationships with avocado roots, effectively controlling soil-borne pathogens through mechanisms such as the production of volatile compounds and competition for resources [31]. Also, *Pseudomonas* can produce siderophores, which are crucial for iron acquisition in soils with iron limitation, consistent with the lower content found in LE [32]. Notably, the family *Pseudomonadaceae* and genus Pseudomonas are particularly abundant in the LE soil, which may have beneficial implications for crop health by contributing to functional traits, including control of root pathogens. These findings suggest that changes in specific macronutrients lead to shifts in key soil bacterial groups, such as *Pseudomonas*. Interestingly, in LE, there is an overrepresentation of other genera with potential beneficial functions for plants. *Cupriavidus* has been reported as a helper of the plant under high copper levels [33]. In addition, genera such as *Burkholderia* and *Acinetobacter* can mitigate abiotic stresses through hormone production, phosphate solubilization, and antagonistic activity against various plant pathogenic fungi [34, 35]. These results support the concept that well-nourished plants tend to recruit beneficial bacteria.

Following the Copiotrophs–Oligotrophs theory, bacterial taxa can be classified based on their nutritional requirements. Copiotrophs are characterized as fast-growing bacteria with high nutritional demands, whereas oligotrophs grow at a slower rate and can outcompete copiotrophs in low-nutrient conditions [36]. Notably, in this study, we observed an overrepresentation of families from oligotrophic phyla, such as Planctomycetota *(Phycisphaeraceae, Pirellulaceae)* and Chloroflexota *(Caldilineaceae)* in CS [37]. On the other hand, there is an overrepresentation of bacterial taxa regarded as copiotrophs, such as Pseudomonadota *(Gammaproteobacteria: Moraxellaceae*, and *Rhodanobacteraceae*) and *Betaproteobacteria (Burkholderiaceae)* in LE [36]. These findings indicate that the establishment of oligotrophic and copiotrophic bacteria in the rhizosphere is influenced by critical soil parameters. However, for most high taxonomic ranks, generalizing life history strategies becomes more challenging, and finer phylogenetic signals are required [36].

In addition, differences in soil conditions had a noteworthy impact on the correlation patterns within the bacterial communities [38]. It has been demonstrated that nutrient concentration can play a pivotal role in shaping interactions among bacteria [39]. In our study, we observed that bacterial communities in landrace avocado rootstock responded to the variations in soils between the different farms in different forms. Notably, the co-occurrence network in the CS farm exhibited a simpler structure compared to the more complex co-occurrence network in the LE farm. In unstressed soils, more complex co-occurrence networks with higher modularity are observed, and this complexity is proposed to enhance the stability of the microbiome under environmental disturbances [40]. These findings demonstrate that soil conditions influence community properties beyond diversity and composition, supporting the investigation of occurrence networks as indicators of environmental quality [41].

Differences in soil conditions between the farms included crucial agronomic variables, which have a substantial impact on the composition and diversity of bacterial communities. Here, a subset of the measured soil factors played a significant role in explaining the differences in rhizosphere bacterial communities. Although the farms share similar management practices, natural soil conditions may lead to differences in key soil parameters. RDA revealed key factors including phosphorus availability, active carbon, and pH. However, other factors such as magnesium, calcium, iron, and aluminum could contribute to the differences between bacterial communities. The CS farm has soil with higher acidity, creating unfavorable conditions for Hass avocado cultivation [8]. Acidic soils can lead to problems such as aluminum toxicity and reduced availability of essential elements, including phosphorus, calcium, and magnesium [8]. These findings corroborate the results of our study, where, in addition to the presence of acidic soil and high aluminum saturation, there was a notable reduction in the availability of nutrients such as calcium and magnesium. These cations are determinants in the establishment and structure bacterial communities and are crucial elements for avocado nutrition [8, 42], and in this study, they are likely playing a pivotal role in explaining the differences between the farms. LE exhibits more than double the levels of Mg and Ca compared to CS, which could be associated with higher avocado production and the recruitment of specific bacterial communities.

Notably, phosphorus levels exhibited significant variations among the farms. Phosphorus is a vital nutrient for plant growth and has emerged as a key factor in which its availability and chemical form can significantly influence the structure, diversity, and composition of bacterial communities [43]. This nutrient fosters microbial functional interaction, thereby enhancing the diversity and abundance of functional genes within the microbial community [44]. Furthermore, low phosphorus soils usually host a higher proportion of oligotrophic taxa, whereas high P soils tend to consist of more copiotroph taxa [44]. Overall, these results highlight how key nutrients such as phosphorus, magnesium, iron, and calcium, can lead to shifts in bacterial communities, as observed between farms.

Functional predictions from our study revealed that the rhizosphere microbiome exhibited an overrepresentation of pathways associated with degradation, while pathways linked to synthesis were less prevalent. This suggests that there is active consumption of rhizodeposits by bacterial populations in the avocado rhizosphere [45]. This analysis also uncovered that the microbiome includes bacteria capable of breaking down complex molecules to make non-conventional carbon sources available for growth, as evident from the overrepresentation of pathways associated with the degradation of aromatic compounds [46]. Notably, the pathways related to aromatic compound degradation were more prevalent in LE compared to CS, potentially indicating more efficient carbon recycling in LE [47]. Additionally, we observed pathways associated with carbohydrate degradation, which may imply the adaptation of bacteria to plant roots and the utilization of carbohydrate-rich rhizodeposits [48]. The higher representation of these pathways in LE suggests a preference for conventional carbon sources and the use of rhizodeposits as a source of carbohydrates [49].

## Conclusion

This research characterized the rhizosphere bacterial communities associated with farms with similar edaphic conditions with two Hass avocado landrace rootstocks. The results revealed shifts in bacterial community properties between the two farms, while no differences were observed between the two avocado rootstock genotypes. Moreover, the dissimilarities observed in this study were linked to differences in crucial soil variables between the two farms, influencing the structure of bacterial communities. This work underscores the importance of soil conditions and how well-nourished plants can recruit a stable microbiome that potentially fulfills beneficial functions, creating a beneficial cycle for the plant. These findings provide valuable insights into the microbiomes associated with commercially important crops such as Hass avocado, and how bacterial communities respond to different soil edaphic conditions. Overall, our study underscores the importance of considering edaphic conditions and soil management strategies for optimizing crop yield and health through microbiome modulation.

## Supplementary Information

Below is the link to the electronic supplementary material.

Supplementary file1 (PDF 2594 KB)

## Data Availability

Raw sequences associated with this study are available at the SRA repository, under BioProject PRJNA742750.

## Code Availability

Codes are available upon request.
