# Supplementary material for: Key Chemical Soil Parameters for the Assembly of Rhizosphere Bacteria Associated with Avocado Cv Hass Grafted on Landrace Rootstocks
Source: Curr Microbiol. 2024 Oct 16;81(12):412. doi: 10.1007/s00284-024-03917-0 (PMC11485190; doi:10.1007/s00284-024-03917-0)
Supplement: Supplementary file 1 — Supplementary file1 (PDF 2594 KB) [file 284_2024_3917_MOESM1_ESM.pdf]

Key chemical soil parameters for the assembly of rhizosphere bacteria associated with avocado cv Hass grafted on landrace rootstocks

Current Microbiology

Mateo Córdoba-Agudelo[1], Juan C. Arboleda-Rivera[1], David A. Borrego-Muñoz[2], Camilo A. Ramírez-Cuartas[1], Juan E. Pérez-Jaramillo[1]*

[1]Instituto de Biología, Universidad de Antioquia, Medellín, Colombia.

[2]Escuela de Microbiología, Universidad de Antioquia, Medellín, Colombia.

*juan.perez@udea.edu.co

Fig. S1 Alpha rarefaction. Alpha diversity Accumulation Curves (Shannon Index) by the depth of sampling after rarefaction, CS (dark blue) shows greater diversity concerning LE (light blue).

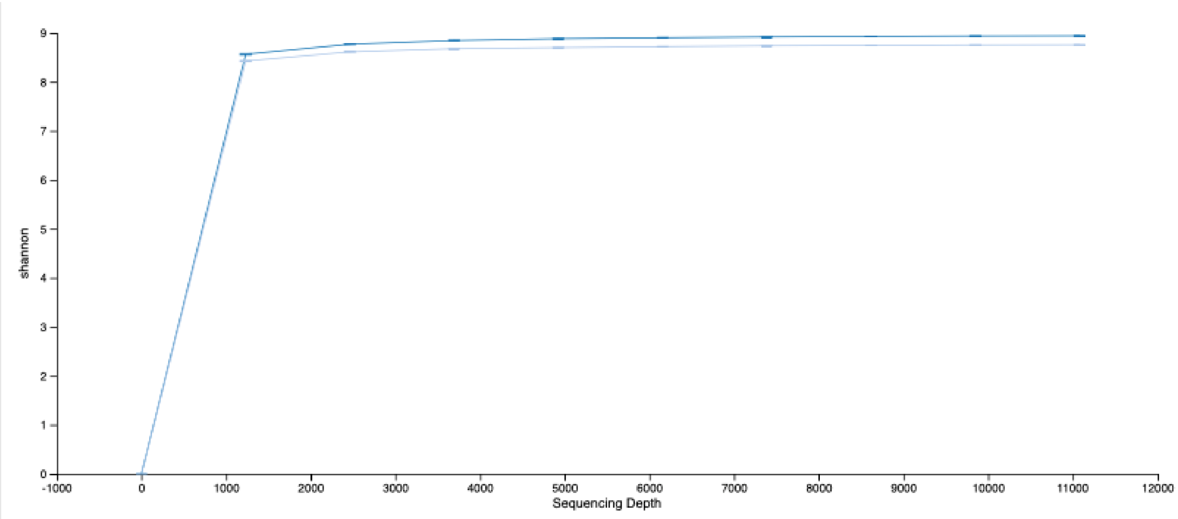

Fig. S2 Alpha diversity of rhizosphere bacterial communities associated with two landraces rootstock genotypes of Hass avocado in two farms. The Chao1, Faith, and Shannon indices were measured using ASVs abundance table. The comparison of indices between farms (left) and genotypes (right) were determined by Kruskall Wallis Test (p<0.05). Each test includes 16 replicates per category. Only the Shannon index had significant differences between farms. No differences between genotypes were detected.

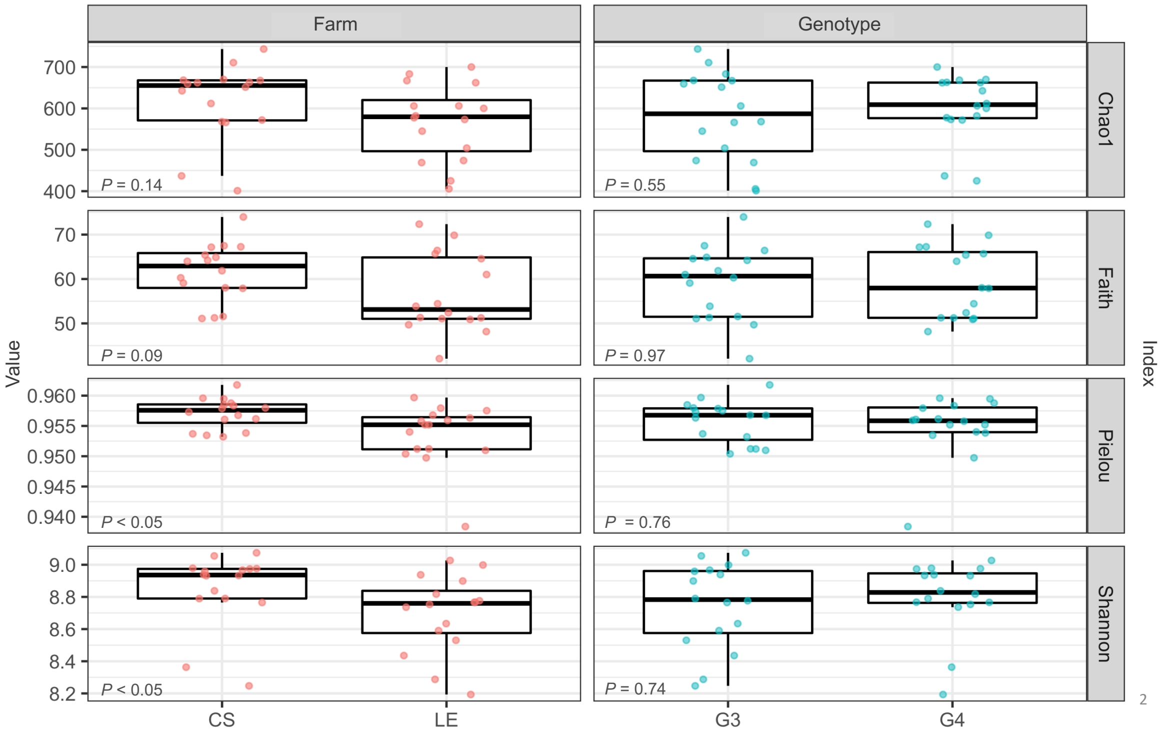

Fig. S3 Beta diversity distances. Bray-Curtis distances and weighted UniFrac within each farm (A) and each genotype (B). The p-value of the PERMANOVAs indicates significant differences between farms (p<0.05) and not among genotypes (p > 0.05).

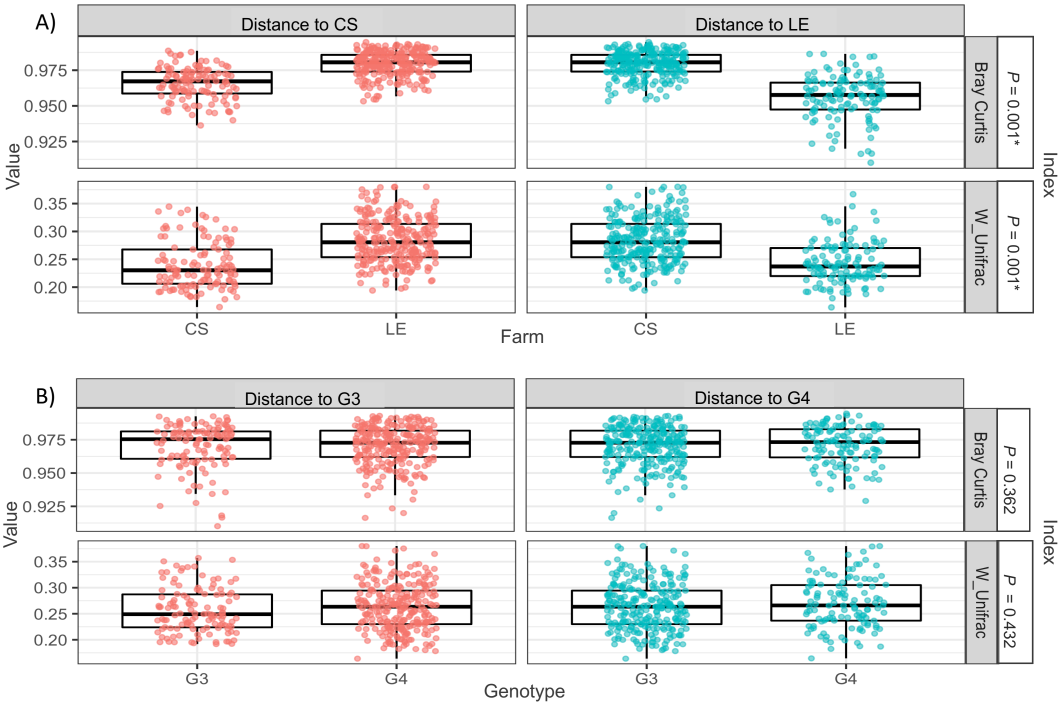

Fig. S4 Constrained Analysis of Principal Coordinates (CAP) based on Bray-Curtis distances utilizing Genotype as the constraining factor. Left: CS farm. Right: LE farm.

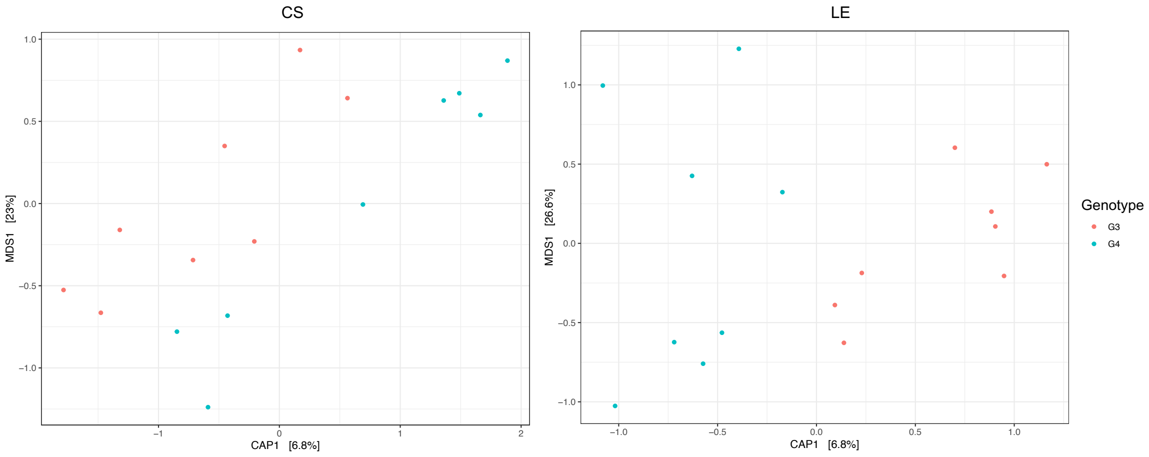

Table S1. Historical climatic and soil variables. Data include climatic variables and variables associated with avocado production and soil. The variables with significant differences between farms are shown in Table 1.

**Variable**	**CS**	**LE**
Average Ambient temperature (°C)	16.08	17.7
Number of avocados produced (Average per tree)	165.83	260.6
Number of avocados exported (Average per tree)	142.63	348.78
Weigth of avocados exported (Average per tree in grams)	30028.66	54669.63
Weigth of avocados produced (Average per tree in grams)	34088.33	42976.27
Count of aerobic endospore forming bacteria	3942547.22	101588.88
Electric conductivity (dS/m)	0.03	0.435
Organic matter (%)	10.63	15.09
Available P (mg/kg)	2.69	5.71
Exchangeable acidity (cmol/kg)	1.18	0.28
Interchangeable Al (cmol/kg)	0.77	0.12
Interchangeable Ca (cmol/kg)	1.14	3.265
Interchangeable Mg (cmol/kg)	0.43	1.05
Available Fe (mg/kg)	312.44	85.3
Available Cu (mg/kg)	1.89	3.83
Ca saturation (%)	0.38	0.65
Mg saturation (%)	0.14	0.21
Al saturation (%)	0.26	0.02
Relative humidity Average (%)	79.49	80.22
Photosynthetically Active Radiation Average (μmol m-2 s-1)	922.69	925.92
Solar radiation Average (nm)	473.54	481.92
Pluviosity (mm/month)	130.44	105.34
Logarithm of total culturable bacteria	8.36	8.79
Logarithm of total culturable *Pseudomonas* spp.	5.51	5.14
pH	5.21	5.35
Avaible S (mg/kg)	7.09	11.14
K interchangeable (cmol/kg)	0.29	0.345
Na interchangeable (cmol/kg)	0.06	0.08
Cation exchange capacity	3.09	5.01
Available Mn (mg/kg)	2.23	2.36
Available Zn (mg/kg)	1	3.11
Available B (mg/kg)	0.25	0.55
K saturation (%)	0.08	0.07
Na saturation (%)	0.017	0.018
Al saturation (%)	0.255	0.018
Ca/Mg	2.82	3.15
Ca_Mg/K	8.53	13.3
Mg/K	2.15	3.22
Ca/B	1047.33	2136.83

Table S2. Per-sample demultiplexed sequence counts. Summary of demultiplexed sequence counts: minimum of 64,436 sequences, median of 75,771, average of 75,818,375, maximum of 82,774, and a total of 2,426,188 sequences across all samples

Farm	sample	Sequence Count
CS	A17	72065
CS	A18	76546
CS	A19	78229
CS	A20	78738
CS	A21	79031
CS	A22	76716
CS	A23	78840
CS	A24	82774
CS	A25	64436
CS	A26	81531
CS	A27	74799
CS	A28	72361
CS	A29	81234
CS	A30	65989
CS	A31	74588
CS	A32	75745
LE	A1	77286
LE	A10	71044
LE	A11	72780
LE	A12	79930
LE	A13	81374
LE	A14	72447
LE	A15	71379
LE	A16	78014
LE	A2	75570
LE	A3	75797
LE	A4	73571
LE	A5	81984
LE	A6	75700
LE	A7	79924
LE	A8	71854
LE	A9	73912

Table S3. Top 30 relative taxa abundance in each condition at family and genus level

Farm	Family	Relative abundance (%)	Genus	Relative abundance (%)
CS	Xanthomonadaceae	0.355829923	Woeseia	0.279637878
CS	Gimesiaceae	0.385392519	Massilia	0.280715548
CS	Streptomycetaceae	0.507625482	Devosia	0.282083911
CS	Mycobacteriaceae	0.516729847	Reyranella	0.283074362
CS	WD2101 soil group	0.59744057	Cloacibacterium	0.298988592
CS	Sulfuricellaceae	0.628780094	Nakamurella	0.306753373
CS	Holophagaceae	0.649046391	Arenimonas	0.309178241
CS	Blastocatellaceae	0.658288893	Streptomyces	0.312196797
CS	Haliangiaceae	0.660611759	Dongia	0.824353345
CS	Phycisphaeraceae	0.721397214	Bryobacter	0.477925289
CS	Caulobacteraceae	0.839312233	Mycobacterium	0.516729847
CS	Micromonosporaceae	0.899135891	Pedomicrobium	0.843755817
CS	Sphingomonadaceae	0.920946891	Flavisolibacter	0.551907413
CS	Flavobacteriaceae	0.928859002	Sphingomonas	0.600984498
CS	Dongiaceae	0.988707317	Chthoniobacter	0.628780094
CS	Solirubrobacteraceae	1.036601557	Haliangium	0.660611759
CS	Verrucomicrobiaceae	1.132311844	Bradyrhizobium	0.708785463
CS	Rhizobiaceae	1.212032031	Terrimonas	0.749065877
CS	Pseudomonadaceae	0.412314262	Hyphomicrobium	0.829036425
CS	Acidobacteriaceae (Subgroup 1)	1.412066209	Comamonas	0.833227816
CS	Ktedonobacteraceae	1.676022864	Pseudomonas	0.880726488
CS	Burkholderiaceae	1.884139193	C. udeaobacter	0.882601324
CS	Acidothermaceae	1.89782379	Ellin6067	0.899135891
CS	Nitrosomonadaceae	1.916590956	Bryobacter	0.928859002
CS	Chitinophagaceae	2.129111561	Gemmatimonas	0.988707317
CS	Gemmatimonadaceae	2.149754442	MND1	1.166528031
CS	Solibacteraceae (Subgroup 3)	2.471348907	Acidibacter	1.668128706
CS	Pedosphaeraceae	2.97752056	Haliangium	1.916590956
CS	Microscillaceae	4.366759082	Acidothermus	1.871348907
CS	Xanthobacteraceae	5.16713458	Candidatus Solibacter	2.581600113
LE	Nitrosomonadaceae	0.30700888	Bacillus	0.242422961
LE	Gimesiaceae	0.339384074	Chthoniobacter	0.247050942
LE	Acetobacteraceae	0.340230463	Pseudolabrys	0.269006929
LE	Streptomycetaceae	0.350712528	Pseudonocardia	0.27292603
LE	Xanthomonadaceae	0.357367309	Devosia	0.300366352
LE	Micropepsaceae	0.38341649	Micromonospora	0.30233482
LE	Caulobacteraceae	0.496842532	Sphingomonas	0.347384392
LE	Dongiaceae	0.515063491	Terrimonas	0.300255527
LE	Flavobacteriaceae	0.540642327	Holophaga	0.412151279
LE	Haliangiaceae	0.586632112	Sphingobium	0.440703701
LE	Micrococcaceae	0.593198659	Paenarthrobacter	0.479358495
LE	Solirubrobacteraceae	0.636636269	Dongia	0.515063491
LE	Rhizobiaceae	0.654280258	Flavobacterium	0.540642327
LE	Blastocatellaceae	0.812707917	Granulicella	0.548799061

LE	Holophagaceae	0.964533677	Haliangium	0.586632112
LE	Verrucomicrobiaceae	1.005752907	Bradyrhizobium	0.646077919
LE	Sulfuricellaceae	1.007687388	Massilia	0.648104386
LE	Sphingomonadaceae	1.10642477	Polaromonas	0.715590153
LE	Pseudomonadaceae	1.144404359	Cupriavidus	0.868572836
LE	Acidobacteriaceae (Subgroup 1)	1.256196607	Roseimicrobium	0.924025625
LE	Ktedonobacteraceae	1.39884538	Ellin6067	0.94324428
LE	Microscillaceae	1.452708703	C. udeaobacter	0.972530359
LE	Nitrosomonadaceae	1.483613036	MND1	1.005752907
LE	Acidothermaceae	2.721263609	Gemmatimonas	1.007687388
LE	Chitinophagaceae	1.862472896	Bryobacter	1.088552136
LE	Gemmatimonadaceae	1.903441276	Acidibacter	1.144404359
LE	Solibacteraceae (Subgroup 3)	3.985373689	Haliangium	1.368358071
LE	Pedosphaeraceae	4.611446019	Acidothermus	2.252708703
LE	Xanthobacteraceae	5.249434832	C. Solibacter	2.316752263
LE	Burkholderiaceae	7.456826344	Pseudomonas	5.249434832
